# Cost-Effectiveness of Timely Surgery and Timely Inpatient Rehabilitation for the Management of Hip Fracture

**DOI:** 10.2106/JBJS.OA.26.00200

**Published:** 2026-09-09

**Authors:** Zheng Jing Hu, Lawrence Mbuagbaw, Gerhard Fusch, Salhab El Helou, Judith Versloot, Lehana Thabane, Walter Wodchis

**Affiliations:** 1 Department of Health Research Methods, Evidence and Impact, McMaster University, Hamilton, Ontario, Canada; 2 Department of Pediatrics, Division of Neonatology, McMaster University, Hamilton, Ontario, Canada; 3 Institute for Health Policy Management and Evaluation, University of Toronto, Toronto, Ontario, Canada; 4 The Research Institute of St Joseph’s Healthcare, Hamilton, Ontario, Canada

## Abstract

**Background:**

Evidence-based and quality-of-care guidelines for hip fracture have recommended providing timely surgery and postacute rehabilitation. We sought to evaluate the cost-effectiveness of providing early surgery (within 24 hours of emergency department admission) and immediate admission to inpatient rehabilitation postacute discharge, or a combination of both for the management of hip fracture patients in Ontario, Canada.

**Methods:**

We used a Markov state transition model to compare early surgery and immediate rehabilitation to surgery beyond 24 hours and no postacute rehabilitation or receiving it beyond 24 hours postacute discharge. Costs and Quality-adjusted life-years (QALYs) were estimated in a 5-year horizon for elderly patients, from the perspective of the (public) insurance payer in Ontario, using linked administrative data sets. Transition probabilities and costs were obtained from regression models fitted using linked administrative data sets. EuroQoL 5-Dimension (EQ-5D) utility values were obtained from an international prospective observational study. We performed base-case and probabilistic sensitivity analysis using 5,000 simulations for willingness-to-pay thresholds from $0-$300,000 Canadian Dollars (CAD).

**Results:**

The incremental cost-effectiveness ratio of timely rehabilitation, for both timely surgery and rehabilitation, compared with neither were $124,162 and $83,714 per QALY, respectively. Timely surgery alone was an absolute advantage strategy (Δ Cost = −$2,387, Δ QALY = 0.06). Five-year base-case costs ranged from $130,747 to $156,848 and QALYs from 2.13 to 2.37. Timely surgery alone had the highest probability of being cost-effective up to a willingness-to-pay threshold of $128,000 per QALY, whereas combined surgery and rehabilitation was favored above $130,000 per QALY. Results were qualitatively similar for common diagnoses and procedure modalities.

**Conclusion:**

Our population data and modelling results indicate that timely surgery can improve patients’ quality of life while reducing costs. Adding inpatient rehabilitation yields greater QALYs but at higher cost.

**Level of Evidence:**

Economic Level IV. See Instructions for Authors for a complete description of levels of evidence.

## Background

Hip fracture is a major contributor of morbidity, mortality, and healthcare burden among older adults, with a direct cost of $60,633 per case in Canada in 2019^1^. Patients with hip fracture have a one-year mortality rate of 20% to 25%^2^, only 30% to 34% return to prefracture functioning^3^, and approximately one-third require permanent care in nursing homes^3^.

The evidence and best practice guidelines for providing good care for patients with hip fracture are well established^4^. Among these are receiving surgery within 24 hours of emergency department (ED) admission and rehabilitation following discharge.

A systematic review by Simunovic et al. showed that receiving timely surgery was associated with significant incremental reduction in mortality, at thresholds of receiving surgery within 72 hours, 48 hours, or 24 hours of patients’ admission to the ED^5^. A propensity-score matched study by Pincus et al. showed that receiving surgery within 24 hours of admission was associated with significant reduction in 30-day mortality (risk difference (RD) 0.79, 95% confidence interval [CI], 0.23-1.35), 1-year mortality (RD 2.31, 95% CI, 1.47-3.25), and the composite outcome of mortality and various medical and surgical complications, at 30 days, 90 days, and 1-year postdischarge^6^.

Numerous randomized controlled trials have also demonstrated the benefits of postacute rehabilitation in improving patients’ mobility and independent functioning. These programs include both in-hospital and home-based physiotherapy and progressive resistance training. Narrative reviews have indicated that both settings of rehabilitation have shown benefits in patient outcomes compared with standard care^7,8^. Inpatient rehabilitation may also yield greater clinical benefits. A Cochrane review of multidisciplinary rehabilitation programs concluded that those taking place in a hospital setting reduced the composite of mortality and deterioration in residential status, whereas the benefits of home-based rehabilitation remained unclear^9^. An observational study comparing home-based and inpatient rehabilitation by Levi et al. showed that patients who underwent inpatient rehabilitation had greater gain in functional independence measure^10^, while another propensity-score matched study by Pitzul et al. showed that patients who received inpatient rehabilitation had lower rates of mortality and hospitalization at 30 days and 1 year following acute care discharge compared with patients who received community-based rehabilitation^11^.

Altogether, the evidence supports receiving surgery within 24 hours of admission to the ED and inpatient rehabilitation following postacute discharge. Notably, however, while rapid surgical access requires process redesign and possibly marginal incremental costs, inpatient rehabilitation also carries a substantially higher cost to the healthcare system^11^. Thus, an important question for healthcare policy, planners, and organizational leaders is whether investing additional resources to achieve timely access to surgery and inpatient rehabilitation would be cost-effective.

We conducted a cost-effectiveness analysis to determine whether timely surgery within 24 hours of ED admission, timely inpatient rehabilitation after acute care discharge, or both are cost-effective strategies for managing patients with hip fracture, compared with neither intervention.

## Methods

We developed a Markov state–transition cohort model to estimate the cost-effectiveness of the 4 management strategies for patients with hip fracture, who are 50 years or older at admission, admitted from the community (i.e. not residing in long-term care before admission), received surgery for hip fracture, and survived their index hospitalization. Model parameters for transition probabilities and costs were obtained from records of individuals admitted to any hospital in Ontario, Canada, while health state utility values were taken from the International Costs and Utilities Related to Osteoporotic Fractures Study^12^. The study cohort consisted of all persons hospitalized for an index hip fracture as a main diagnosis (i.e. contributed the most to hospital length of stay), between April 1, 2015, to March 31, 2019, with International Classification of Diseases, Tenth Revision (ICD-10) diagnoses codes S72.0, S72.1, and S72.2, who received a hip fracture surgery during their hospital stay and were discharged alive; the description of this cohort is shown in Table I. We also conducted subgroup analysis for intertrochanteric fractures with pinning fixation, femoral neck fractures with partial arthroplasty and pinning fixation, and femoral neck fractures with full arthroplasty.

**Table I:** Descriptive Statistics of the Demographic Characteristics and Outcomes of the Hip Fracture Study Cohort, by Management Strategy

Demographic Characteristics and Outcomes	No Timely Surgery, No Timely Rehabilitation	Timely Rehabilitation, No Timely Surgery	Timely Surgery, No Timely Rehabilitation	Timely Surgery and Timely Rehabilitation
Total Number of patients, n (%)	8,139 (23.0)	12,659 (35.7)	6,744 (19.0)	7,903 (22.3)
Age, mean (SD)	79.4 (11.6)	81.9 (9.6)	77.4 (12.2)	81.5 (9.9)
Female sex, n (%)	5,326 (65.4)	8,656 (68.4)	4,573 (67.8)	5,693 (72.0)
Frailty score mean (SD)	7.1 (5.3)	6.6 (4.1)	5.6 (4.5)	5.8 (3.7)
Number of active comorbidities, mean (SD)	5.13454 (2.5)	5.4 (2.4)	4.5 (2.4)	5.0 (2.3)
Recurrence of hip fracture, n (%)	538 (6.6)	1,320 (10.4)	443 (6.6)	857 (10.8)
Time to recurrence of hip fracture, days, mean (SD)	850.3 (527.2)	877.3 (521.0)	911.9 (514.2)	881.5 (518.8)
Mortality occurring during observation period, n (%)	3,628 (44.6)	4,436 (35.0)	2,363 (35.0)	2,406 (30.4)
Time to death, days, mean (SD)	342.3 (398.0)	548.9 (431.5)	413.2 (426.0)	566.6 (434.4)
Total days observed, mean (SD)	706.0 (523.8)	842.9 (475.1)	813 (515)	885 (473)
Total observed cost, mean (SD)	$84,815 (88,922)	$115,988 (96,489)	$76,289 (84,896)	$106,814 (91,645)
Femoral, n (%)	4,444 (54.6)	6,255 (49.4)	3,555 (52.7)	3,744 (47.4)
Intertrochanteric, n (%)	3,248 (39.9)	5,659 (44.7)	2,800 (41.5)	3,685 (46.6)
Subtrochanteric, n (%)	447 (5.5)	745 (5.9)	389 (5.8)	474 (6)
Full, n (%)	923 (11.3)	1,273 (10.1)	680 (10.1)	787 (10)
Partial, n (%)	2,311 (28.4)	3,757 (29.7)	1,675 (24.8)	2,168 (27.4)
Pinning, n(%)	4,698 (57.7)	7,353 (58.1)	4,251 (63)	4,791 (60.6)
Other, n (%)	207 (2.5)	276 (2.2)	138 (2)	157 (2)

We modeled the effectiveness of timely surgery and inpatient rehabilitation through regression models that predicted the probability of transitioning to each health state, by including timely surgery and inpatient rehabilitation as covariates in these models. Transition probabilities were derived from logistic and survival regression models and costs of each health state through a gamma link function. Internal and external validation assessed how well these estimates implied counterfactuals.

All data holdings are housed at ICES (formerly Institute for Clinical Evaluative Sciences)^13^. These administrative data sets, which excludes racial identifiers, otherwise contain all the variables used to model transition probabilities and costs, including age, sex assigned at birth, comorbidities, frailty, and the date and type of fragility fracture. The analysis was conducted on a 5-year horizon, from the payer perspective of the Ontario Ministry of Health and Long-Term Care, in 2019 Canadian Dollars, and a discount of 3.5% was applied to costs and to quality-adjusted life-years (QALYs)^14^.

### Economic Modelling

**Fig. 1:**
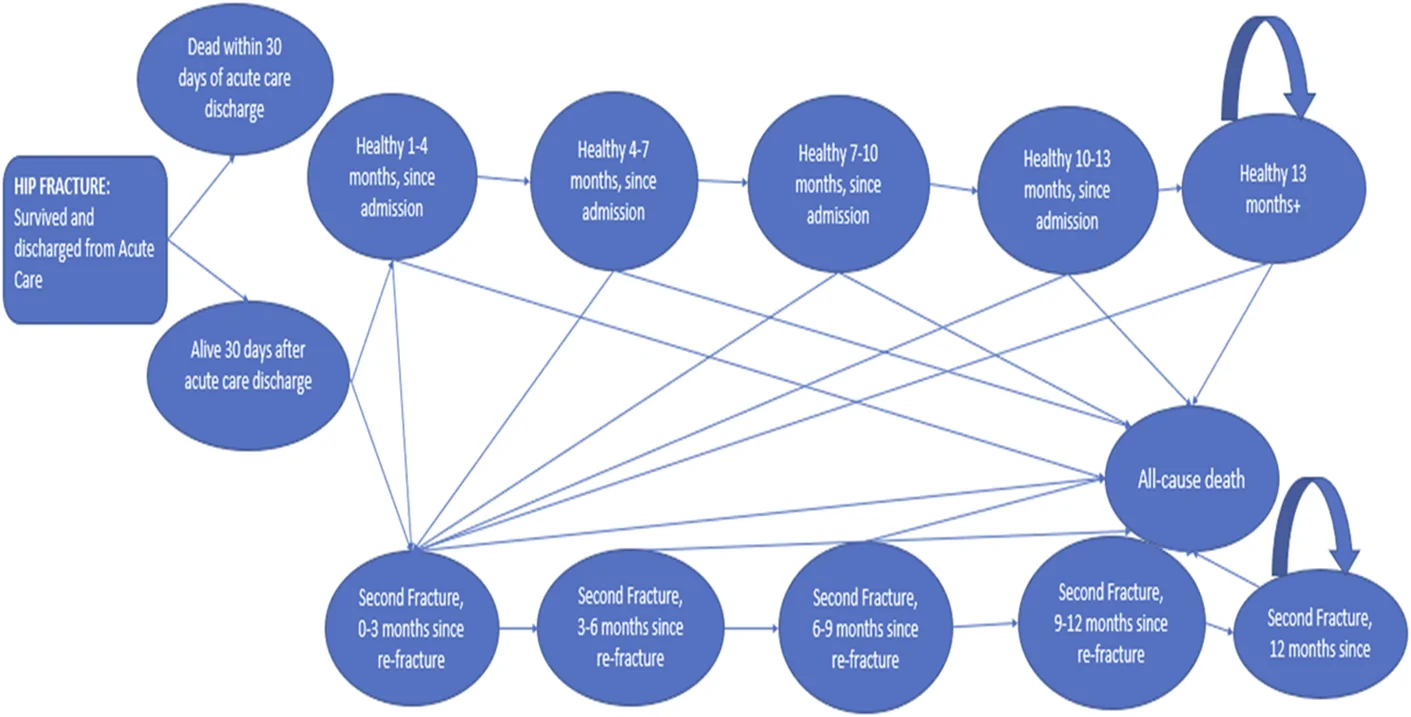
Structure of the Markov Model.

Details of our Markov economic model, including subgroup analyses description and results, are elucidated in Appendices A, A2, B, and C^5,6,9,11,12,15-25^. Figure 1 shows the model diagram.

## Results

### Cohort Description

Our observational cohort consisted of 35,445 hip fracture patients. Women consisting of 68% of patients, and the average age was 80 years at the time of admission. The largest group of patients belonged to the treatment group of receiving timely inpatient rehabilitation alone (36.4%), and the other groups were evenly distributed. There was a sizable difference in the age and frailty distribution between groups. Patients who received timely surgery alone tended to be younger patients with lower frailty, whereas patients who received timely rehabilitation alone were older and frailer. Interestingly, there was a higher rate of second fragility fracture among patients who received inpatient rehabilitation, possibly attributed to these patients being frailer or surviving longer postdischarge (Table I).

### Cost-Effectiveness Analysis

When considering the base-case costs of healthcare services accrued from a public payer perspective, compared with neither receiving timely surgery nor timely inpatient rehabilitation, the incremental cost-effectiveness ratio of both timely surgery and timely rehabilitation and timely rehabilitation alone, compared with neither were $124,162 and $83,714 per QALY. Receiving surgery within 24 hours of admission to the ED alone was an absolute advantage strategy, both saving costs and increasing QALYs (Δ Cost = −$2,387, Δ QALY = 0.06). The 5-year modeled costs for an 80-year-old woman with a frailty score of 6.3 and 5 active chronic conditions were $133,134, $156,868, $130,747, and $153,224 for neither strategy, timely inpatient rehabilitation alone, timely surgery alone, and both strategies, respectively. The respective QALYs were 2.13, 2.32, 2.19, and 2.37 (Table II).

**Table II:** Predicted Costs and Quality-Adjusted Life-Years Accrued From a Public Payer Perspective Over 5 Years Following Hip Fracture and Incremental Cost Effectiveness Ratio, by Base-Case and Sensitivity Analysis*

Strategy	QALY (5 Years)	Cost (5 Years)	QALY Difference (vs. None)	Cost Difference (vs. None)	Incremental Cost-Effectiveness Ratio
Base-case results					
No timely surgery, No timely rehabilitation	2.13	$133,134			
Timely surgery, no timely rehabilitation	2.19	$130,747	0.06	−$2,387	Dominant
Timely rehabilitation, no timely surgery	2.32	$156,868	0.19	$23,733	$200,930.77
Timely surgery and timely rehabilitation	2.37	$153,224	0.24	$20,089	Dominant
Sensitivity analysis results- inpatient rehabilitation within 7 days					
No timely surgery, No timely rehabilitation	2.13	$131,867			
Timely surgery, no timely rehabilitation	2.19	$129,515	0.06	−$2,353	Dominant
Timely rehabilitation, no timely surgery	2.32	$157,796	0.19	$25,928	$217,546.15
Timely surgery and timely rehabilitation	2.37	$154,148	0.24	$22,280	Dominant

QALY = quality-adjusted life-years.

* Costs and QALYs accrued assuming that the person is an 80-year-old woman with a frailty score of 6.3 and 5 active chronic conditions, without any history of falls or home care.

**Fig. 2:**
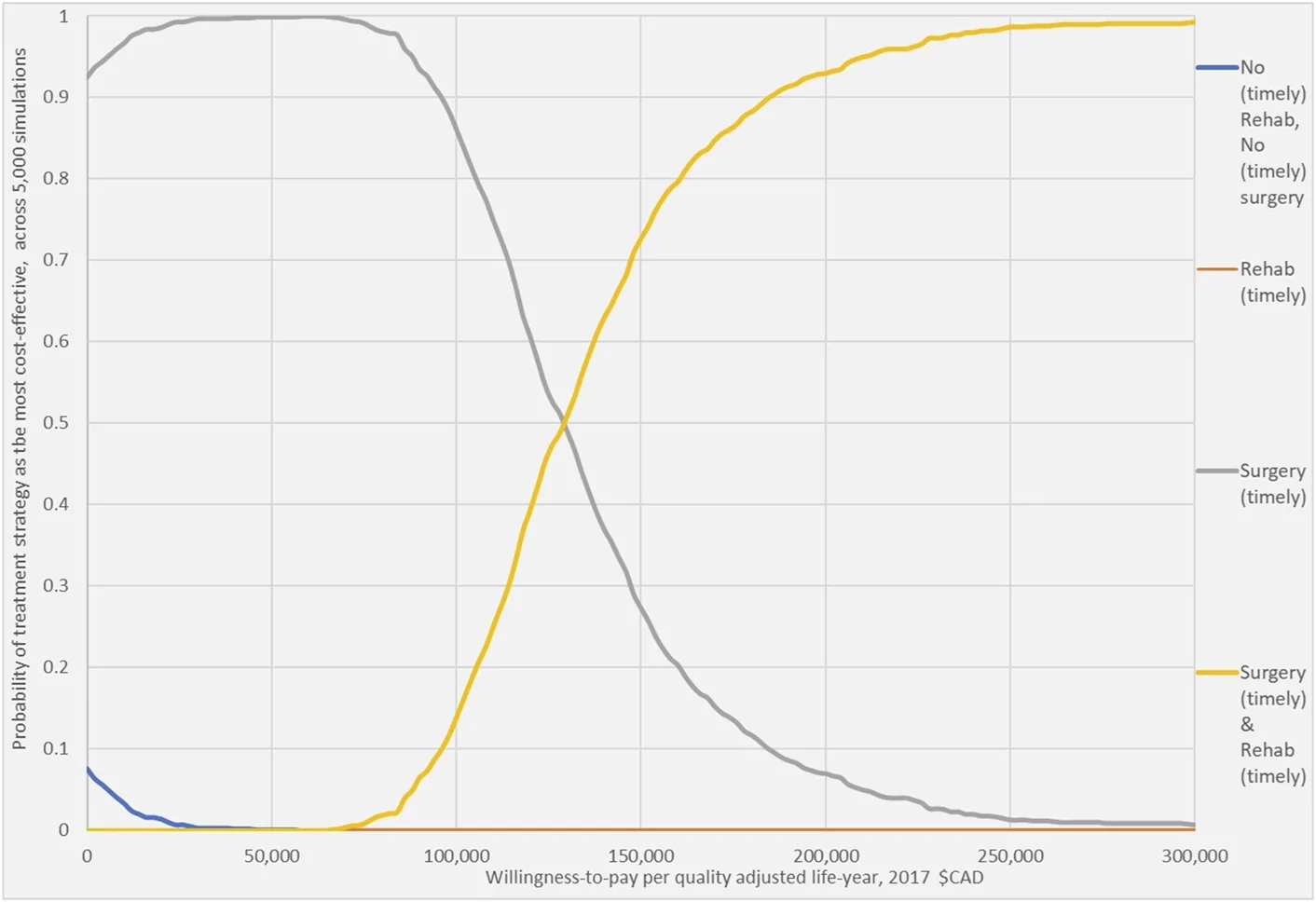
Cost-effectiveness acceptability curve: probability of each strategy as most cost-effective at various willingness-to-pay thresholds.

In probabilistic sensitivity analysis, the QALYs for 5,000 simulations ranged from 1.2 to 3 and modeled 5-year costs ranged from $90,000 to $250,000. Timely surgery alone was the optimal strategy for a willingness-to-pay threshold below $128,000, and achieving both timely surgery and timely rehabilitation was the optimal strategy above $130,000 per QALY (Fig. 2).

**Fig. 3:**
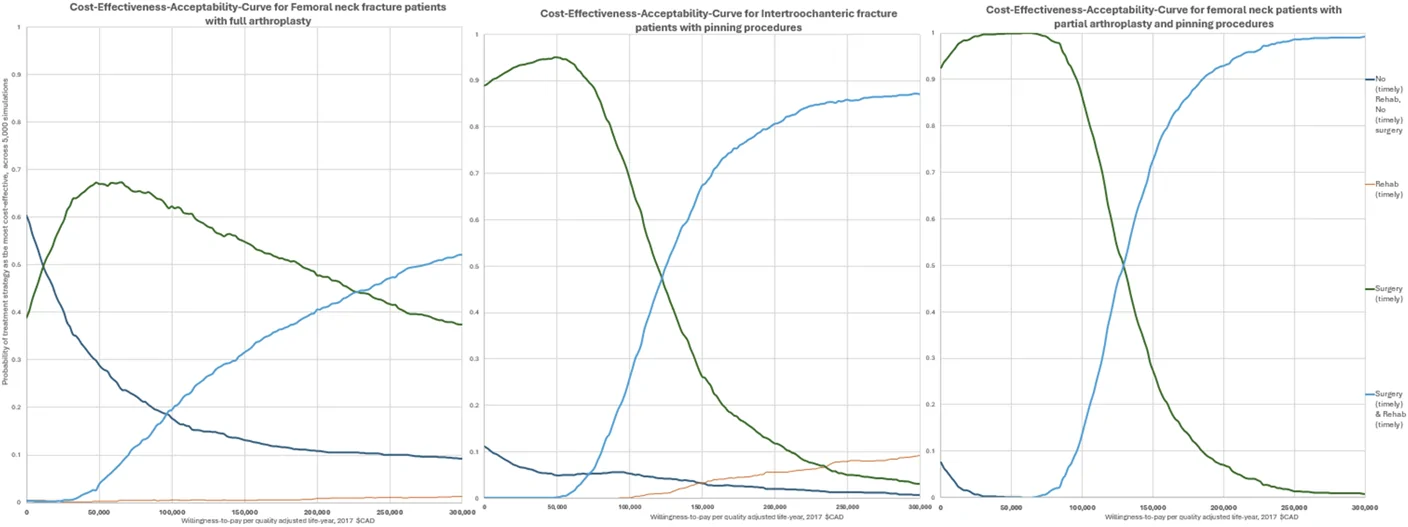
Cost-effectiveness acceptability curve: probability of each strategy as most cost-effective at various willingness-to-pay thresholds, among the 3 subpopulations.

When the definition of receiving timely inpatient rehabilitation was changed to be within 7 days instead of same day or next day, QALYs and costs only had minor changes (Table II). Model assessment and validation results are shown in Appendices D, E, and F. In subgroup analyses, timely surgery is the most cost-effective strategy beyond $10,000 per quality-adjusted life-years (Fig. 3) for femoral full arthroplasty patients and dominant in other subgroups.

## Discussion

The study found that on average, providing timely surgery is a cost-saving strategy from the public payer perspective. While combined timely surgery and inpatient rehabilitation offer a greater improvement in quality of life, such a strategy comes with a much higher cost for the healthcare payer. The results of sensitivity analyses involving 7-day admission for inpatient rehabilitation, and subpopulations, showed timely surgery remaining as cost-saving or the most cost-effective within acceptable willingness-to-pay thresholds.

Interestingly, our study found a higher rate of second fragility fracture among patients who received inpatient rehabilitation, even after adjusting for prognostic factors. This finding contrasts a previous study showing that patients who underwent inpatient rehabilitation had lower rates of all-cause mortality and rehospitalization 1 year after hip fracture^26^. Our findings may reflect unmeasured confounders, including polypharmacy^27^, and limitations of capturing disease severity^28^, which are incompletely captured in administrative data. Furthermore, patients who did not receive inpatient rehabilitation may still have received home-based rehabilitation. Another study from Ontario showed that patients discharged directly home received home-based rehabilitation at about twice the rate of those discharged home after inpatient rehabilitation^29^. Patients completing inpatient rehabilitation may return to more independent living, with greater mobility and, thus, fall exposure. These differences in care pathway and exposure may explain the higher observed second-fracture rate in the inpatient rehabilitation group without implying a causal effect of inpatient rehabilitation. Beyond surgery, improvements in quality of life may provide considerations for access and use of inpatient rehabilitation for specific populations.

While achieving surgery within 24 hours for the average patient has shown to be cost-effective at all willingness-to-pay thresholds, the addition of inpatient rehabilitation only becomes more likely to be cost-effective at nearly $130,000 CAD per QALY or above. Although this exceeds $50,000 willingness-to-pay per QALY^30^, in Canada, decisions to adopt or reject an intervention also accounts for efficacy, effectiveness, burden of disease, equity, ethics, and other key public health decision criteria^31^.

Although patients receiving inpatient rehabilitation have higher QALYs compared with those without, home rehabilitation may suffice for patients with lower severity hip fracture. Studies have reported that patients who receive home rehabilitation achieved similar or better patient-reported outcomes as inpatient rehabilitation^10,32^. Future research should directly assess the benefits of inpatient vs. home rehabilitation for subgroups of patients using the Functional Independence Measure^33^ recorded at admission and discharge^34^ or InterRAI assessments^35-37^. More patient data and artificial-intelligence based prediction may offer future value to select patients for high-cost inpatient rehabilitation.

This study does not mandate physicians to perform surgery within 24 hours against clinical and safety factors. Numerous clinical factors require delayed surgery beyond 24 hours, including anticoagulation medications^38^, cardiac and cardiovascular conditions^39^, need for diagnostic imaging^40^, or medical stabilization^40^ before surgery. Instead, this study provides evidence to prioritize system-level changes to reduce avoidable delays. Moreover, the cost-saving benefits of surgery within 24 hours may be limited by the fact that only persons with sufficient physiological reserve receive timely surgery, and some of the cost savings may be driven by unmeasured confounders.

The present analysis is unable to measure individual clinical indications and contraindications for timely surgical intervention. Variations of important clinically indicated delays (e.g., anticoagulation reversal, infection treatment, cardiac stabilization, and anemia correction^40^) are likely to significantly influence the selection of surgical and rehabilitation strategies, ultimately resulting in differing prognostic outcomes for individual patients. Similarly, different surgical procedures are associated with differential inherent risks in anesthesia, postoperative complications, and optimal rehabilitation pathways. Despite this, our subgroup analyses showed that timely surgery remains a cost-saving strategy from the public payer perspective or a preferred strategy for acceptable willingness-to-pay thresholds.

We did not account for caregiver burden or associated productivity losses. Quantifying loss of productivity may indicate inpatient rehabilitation being more cost-effective at a lower willingness-to-pay threshold, through reduced caregiver burden. Second, we could not incorporate other quality indicators into our model that were unavailable or incomplete in our administrative data sets, such as load-bearing-as-tolerated, daily mobilization, screening for or managing delirium, and medication factors that may affect patients’ prognosis^4^, instead, considering them as random variation. Our model also did not include the effect of other comorbidities on health state utility. The available health utility decrements for hip fracture are not specific to concurrent chronic conditions, type of hip fracture, or surgical procedure modality^12,15,41^. We did not examine other potentially impactful outcomes such as postoperative delirium, cardiovascular complications, and infections, which impact individual patient outcomes. We assume these are represented in the original study populations from which our quality-of-life parameters are drawn and are intermediate effects captured in overall utility scores.

Finally, this study, by design, focuses on population averages and system-level interventions that impact hip fracture patients at a population-health level. The probabilities therein reflect population averages, even though individual recovery may vary widely. The study does not assess individuals for whom early surgery may introduce incremental risk. These considerations are paramount for individual clinical decision making. Additional studies with access to data on prefracture function, cognitive impairment, and surgical contraindications may further elucidate subpopulations that may benefit most from these interventions. Ultimately, these findings highlight the need to reduce avoidable surgical delays while sustaining a comprehensive, patient-centered approach to postdischarge orthogeriatric care. They are intended to inform, not override, clinical judgment, which must remain guided by individual patient characteristics, fracture type, and postacute needs of individual patients.

A major strength of our study is the use of real-world administrative data from a large patient cohort over an extended period, to evaluate the effectiveness of timely surgery and timely rehabilitation, as implemented in practice. This provides decision makers with relevant evidence on the cost-effectiveness of timely surgery and inpatient rehabilitation.

## Conclusion

From a population-average public payer perspective in the province of Ontario, Canada, receiving surgery within 24 hours of ED admission for hip fracture is a cost-saving strategy that improves quality of life. Adding inpatient rehabilitation provides modest additional benefit at substantially higher cost and is cost-effective only at higher willingness-to-pay thresholds. These findings support efforts to reduce avoidable surgical delays from a policy decision maker’s perspective, adopt a patient-centered approach to rehabilitation while maintaining individualized clinical decision making, and identify patient subgroups most likely to benefit from inpatient rehabilitation.

## Appendix

Supporting material provided by the authors is posted with the online version of this article as a data supplement at jbjs.org (https://links.lww.com/JBJSOA/B320). This content was not copyedited or verified by JBJS.
